# Mavoglurant in Fragile X Syndrome: Results of two open-label, extension trials in adults and adolescents

**DOI:** 10.1038/s41598-018-34978-4

**Published:** 2018-11-19

**Authors:** Randi Hagerman, Sebastien Jacquemont, Elizabeth Berry-Kravis, Vincent Des Portes, Andrew Stanfield, Barbara Koumaras, Gerd Rosenkranz, Alessandra Murgia, Christian Wolf, George Apostol, Florian von Raison

**Affiliations:** 1 MIND Institute and Department of Pediatrics, UC Davis Medical Center, Sacramento, CA USA; 2 Centre Hospitalier Universitaire Vaudois, Lausanne, Switzerland; 3 CHU Sainte-Justine Research Centre, Montreal, Canada; 4 Rush University Medical Centre, Department of Pediatrics, Neurological Sciences, and Biochemistry, Chicago, IL USA; 5 National Reference Center for Fragile X and Other XLID, CIC 1407 INSERM - Hospices Civils de Lyon, Université de Lyon and CNRS UMR 5304 (L2C2), Bron, France; 6 Patrick Wild Centre, Division of Psychiatry, University of Edinburgh, Royal Edinburgh Hospital, Edinburgh, UK; 7 Neurodegeneration Global Development, Novartis Pharmaceuticals Corporation, East Hanover, NJ USA; 8 Neuroscience Development, Novartis Pharma AG, Basel, Switzerland; 9 Laboratory of Molecular Genetics of Neurodevelopment, Department of Women’s and Children’s Health, University of Padova, Padova, Italy; 10 Lycalis sprl, Brussels, Belgium

## Abstract

Fragile X syndrome (FXS) is the most common monogenic cause of inherited intellectual and developmental disabilities. Mavoglurant, a selective metabotropic glutamate receptor subtype-5 antagonist, has shown positive neuronal and behavioral effects in preclinical studies, but failed to demonstrate any behavioral benefits in two 12-week, randomized, placebo-controlled, double-blind, phase IIb studies in adults and adolescents with FXS. Here we report the long-term safety (primary endpoint) and efficacy (secondary endpoint) results of the open-label extensions. Adolescent (n = 119, aged 12–19 years) and adult (n = 148, aged 18–45 years) participants received up to 100 mg bid mavoglurant for up to 34 months. Both extension studies were terminated prematurely due to lack of proven efficacy in the core studies. Mavoglurant was well tolerated with no new safety signal. Five percent of adults and 16.9 percent of adolescents discontinued treatment due to adverse events. Gradual and consistent behavioral improvements as measured by the ABC-C_FX_ scale were observed, which were numerically superior to those seen in the placebo arm of the core studies. These two extension studies confirm the long-term safety of mavoglurant in FXS, but further investigations are required to determine whether and under which conditions the significant preclinical results obtained with mGluR5 inhibition can translate to humans.

## Introduction

Fragile X syndrome (FXS) is the most common monogenic cause of inherited intellectual and developmental disabilities and has an estimated prevalence of approximately 1 in 4,000 males and 1 in 8,000 females^1,2^. FXS is typically caused by an unstable trinucleotide (CGG) repeat expansion within the promoter region of the fragile X mental retardation 1 gene (*FMR1*), leading to partial or complete loss of the fragile X mental retardation protein (FMRP)^3,4^. The FXS phenotype varies but often includes symptoms of anxiety, perseverative behavior, attention-deficit/hyperactivity disorder (ADHD), seizures, self-injurious behavior, hyperarousal, hypersensitivity, aggression, impaired sleep, and impaired cognition^5–7^. These symptoms are associated with a considerable burden on patients and their caregivers from both a health-related quality of life and economic perspective^8–10^.

There are currently no approved curative therapies for FXS and management approaches focus on symptomatic treatment of comorbid behavioral problems and psychiatric diagnoses (e.g., stimulants for ADHD or selective serotonin reuptake inhibitors [SSRIs] for anxiety disorders associated with FXS). Recent advances in our understanding of the neurobiological basis of FXS have led to the early development and clinical trials of several new potential targeted therapies for FXS^11^.

FMRP regulates mRNA translation and protein production at the synapse via multiple synaptic receptors that include the Group I metabotropic glutamate receptors (mGluR). Loss of FMRP leads to abnormalities of synaptic transmission and dendritic spine architecture that are thought to underlie the behavioral and cognitive symptoms of FXS^12^. Mavoglurant (AFQ056) is a selective mGluR5 antagonist that inhibits activation of the mGluR5 receptor thus reducing the synaptic defects occurring due to absence of FMRP^13^. Preclinical studies have shown that mavoglurant rescues the dendritic spine architecture and restores social behavior in an FXS mouse model (*FMR1* knockout mice)^14,15^. A small-scale phase IIa, placebo-controlled clinical trial of 30 adult male patients with FXS suggested efficacy of mavoglurant in the subpopulation of patients with a fully methylated *FMR1* gene^16^, which supported a larger-scale development program.

Mavoglurant was evaluated as treatment for the behavioral symptoms of FXS in two 12-week, multinational, randomized, double-blind, placebo-controlled Phase IIb clinical studies in adolescent and adult patients (NCT01357239 and NCT01253629, respectively). These studies failed to meet their primary objective of showing mavoglurant efficacy versus placebo in reducing the Aberrant Behavior Checklist – Community edition Fragile X Syndrome specific algorithm (ABC-C_FX_) total score over 12 weeks of treatment in the fully methylated group of patients, or secondarily in the partially methylated or combined groups^17^. Key secondary efficacy objectives, including Clinical Global Impression – Improvement (CGI-I) assessment, were also not significantly different for mavoglurant versus placebo^17,18^. However, mavoglurant was found to be generally safe and well tolerated over this 12-week period.

There are a number of potential reasons why mavoglurant was not found to be effective in these studies. Among these is the relatively brief intervention period in the context of a lifelong severe neurodevelopmental disorder. Two open-label, extension studies in adolescent and adult patients with FXS were initiated as part of the ongoing Phase IIb program to ensure that the trial participants had continued access to the study drug. These extension studies allowed us to further evaluate the safety and efficacy profile of mavoglurant over a longer treatment period.

## Methods

### Study design

These studies were multinational, multicenter, single-arm, flexible-dose, open-label, phase IIb extension of core studies conducted in adolescent (NCT01433354; September 13, 2011) and adult (NCT01348087; May 5, 2011) patients with FXS. The total duration of each extension study was planned to be 24 months or until the study drug would have become available on the market (whichever would have occurred later). The protocol also provided for extension of treatment beyond 24 months, if required. The primary objective of both studies was to evaluate the long-term safety of mavoglurant in these two populations, while secondary objectives included evaluation of its long-term efficacy.

The study protocols were approved by the Independent Ethics Committee or Institutional Review Board (detailed in Tables S1 and S2) at each center. The studies were conducted according to the ethical principles of the Declaration of Helsinki. For both the adolescent and adult studies, the caregiver, legal guardian or legally acceptable representative had to be able to communicate well with the investigator and to understand and support the study requirements by providing written informed consent. If a patient was capable of doing so, he/she should have provided his/her written assent (in accordance with local ethical/regulatory requirements).

### Participants

Eligible participants were males or females who had previously participated in a clinical study of mavoglurant in an FXS population. They should be 12–17 years (adolescent study) and at least 18 years of age (adult study) at the time of screening into the previous study, or at least 12 years of age at the time of entry in the current adolescent study. Participants were required to have a caregiver(s) who spent at least six hours per day with them to supervise treatment and assess outcomes. Major exclusion criteria included a history and/or presence of: any advanced, severe or unstable disease; schizophrenia; bipolar disorder; psychosis; confusional states and/or repeated hallucinations; clinically significant ECG abnormalities; seizure disorder uncontrolled or resistant to therapy within the past 2 years; suicidal behavior or considered to have a high suicidal risk; and severe self-injurious behavior. Patients who used potent inhibitors or inducers of CYP3A4 ≤6 weeks before the extension study baseline were also excluded.

### Treatment

All patients initiated mavoglurant treatment at a starting dose of 25 mg bid and were up-titrated at weekly intervals to 50 mg bid, 75 mg bid, and 100 mg bid (Fig. S1). Dose adjustments were permitted to manage tolerability and to ensure that patients reached their highest tolerated mavoglurant dose, not to exceed 100 mg bid. Patients unable to tolerate the 25-mg bid dose were discontinued. Concomitant medication to treat comorbid behavioral symptoms was permitted but was to be maintained at stable doses during the study as much as possible. Glutamatergic drugs, lithium, warfarin, digoxin, moderate or strong inducers or inhibitors of CYP3A4, and any other investigational drugs were not permitted.

The dosing regimen was to be overseen and documented by the caregiver, where appropriate, to ensure that it was correctly followed.

### Outcomes

#### Safety

Safety assessments involved collection of reports on all adverse events (AEs) and serious adverse events (SAEs), standard clinical laboratory evaluations, and electrocardiograms (ECGs), as well as regular physical examination and assessment of vital signs, body height and weight, and pregnancy status.

#### Behavioral measures and clinical impression

ABC-C_FX_: ABC-C assesses problem behaviors of children and adults with developmental disabilities at home using a 58-item questionnaire completed by the caregiver scoring each item from 0 (“not at all a problem”) to 3 (“problem is severe in degree”)^19,20^. The modified ABC-C_FX_ represents a re-factored 55-item version of the scale based on ratings from a large FXS population and consists of six subscales (irritability, lethargy/withdrawal, stereotypic behavior, hyperactivity, inappropriate speech, and social avoidance), and the total score is graded from 0 to 165^21^.

Clinical Global Impression (CGI) assessment: Baseline severity of illness was assessed using the 7-point CGI-S scale (from “normal” to “among the most extremely ill patients”). Additionally, global changes in symptoms (CGI-I) were rated on a 7-point scale (from “very much improved” to “very much worse”) relative to baseline at entry into the extension study based on clinical evaluation and caregiver reports^22^.

Repetitive Behavior Scale – Revised (RBS-R): RBS-R is a caregiver-rated questionnaire assessing repetitive behaviors across six domains (ritualistic behavior, sameness behavior, stereotypic behavior, self-injurious behavior, compulsive behavior, and restricted interests)^23^. Each domain is scored from 0 (behavior does not occur) to 3 (behavior occurs and it is a severe problem) with a resulting total score between 0 and 129.

Social Responsiveness Scale (SRS): This scale identifies the presence and assesses the extent of autistic spectrum symptoms^24^. It is a 65-item questionnaire, rating behaviors from 0 (not true) to 3 (almost always true) completed by the caregiver, with a total score ranging from 0 to 195.

For all of these scales, a negative change from baseline indicates improvement.

### Statistical analysis

Variables were summarized using descriptive statistics. The number of participants enrolled was determined by the number of eligible patients and not based on power calculations. Patient disposition and efficacy outcomes were analyzed using the “full analysis set,” which included all patients who received at least one dose of study medication during the extension study.

Safety was analyzed using the “safety set,” which consisted of all patients who received at least one dose of study medication during the extension study and had at least one safety assessment occurring after the first dose of extension study medication.

### Role of funding source

The funder of the study was involved in the design and conduct of the study; collection, management, analysis, and interpretation of the data; preparation, review, or approval of the manuscript; and decision to submit the manuscript for publication. The corresponding author had full access to all the data in the study and had final responsibility for the decision to submit for publication. All authors have approved the final version of the paper.

## Results

### Baseline characteristics and drug exposure

The adolescent extension study was conducted between November 23, 2011 and September 17, 2014, whereas the adult extension study was conducted between August 19, 2011 and September 10, 2014.

**Figure 1:**
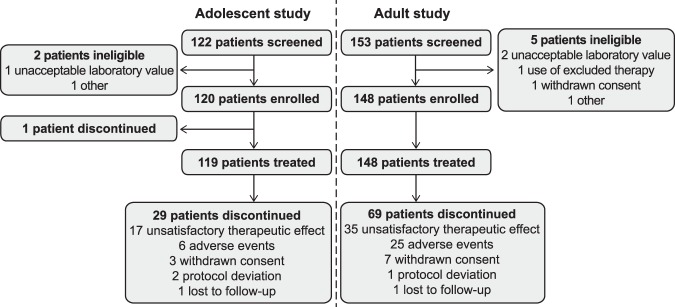
Patient flow*. *The discontinuations noted in this graph are those discontinuations that occurred before study termination. All patients eventually discontinued when the studies ended prematurely.

In total, 119 out of 139 (86%) adolescents and 148 out of 175 (85%) adults entered in the extension studies from their respective double-blind, core studies (Fig. 1)^17^. Baseline demographics and characteristics for both studies are summarized in Table 1.

**Table 1:** Demographics and extension study baseline characteristics (full analysis set).

Demographic variable	Adolescent study (N = 119)	Adult study (N = 148)
Age (years)	15.2 (1.8)	26.6 (6.9)
Range	12–19	18–45
Sex, male, n (%)	106 (89.1)	138 (93.2)
Race, n (%)		
Caucasian	110 (92.4)	142 (95.9)
Black	2 (1.7)	2 (1.4)
Asian	3 (2.5)	0
Native American	1 (0.8)	0
Other	3 (2.5)	4 (2.7)
Weight (kg)	64.5 (16.9)	80.9 (17.9)
Range	34.8–122.5	41.4–131.0
ABC-CFX total score	49.8 (29.0)	43.4 (26.8)
Range	6 to 146	2 to 134
CGI-S score, n (%)		
2 (Borderline mentally ill)	0	0
3 (Mildly ill)	5 (4.2)	3 (2.0)
4 (Moderately ill)	54 (45.4)	64 (43.2)
5 (Markedly ill)	43 (36.1)	55 (37.2)
6 (Severely ill)	17 (14.3)	22 (14.9)
7 (Among the most extremely ill)	0	4 (2.7)
IQ score	41.0 (9.3)	47.0 (7.0)
Range	30–76	36–69

Data are mean (standard deviation [SD]) unless indicated otherwise.

ABC-C_FX_, Aberrant Behavior Checklist – Community edition using the FXS specific algorithm; BMI, body mass index; CGI-S, Clinical Global Impression – Severity scale; IQ, Intelligence Quotient.

Both extension studies were terminated prematurely based on a failure to show efficacy for mavoglurant versus placebo in the core studies, and all patients discontinued. The majority of discontinuation was due to early termination of the studies (Fig. 1).

Mean ± standard deviation (SD) exposure time to mavoglurant in the adolescent study was 513.2 ± 274.94 days, equivalent to 167.2 patient-years. Mean ± SD exposure time in the adult study was 538.3 ± 316.23 days, equivalent to 218.1 patient-years. Overall, 91 patients (76.5%) in the adolescent study and 135 (91.2%) in the adult study received the intended mavoglurant 100 mg bid dose for ≥1 day. In both studies, the overall proportion of patients receiving mavoglurant 100 mg bid remained >69% from Day 22–28 onward. All treated patients were included in the “full analysis sets” and “safety sets” for analysis.

### Safety and tolerability

The majority of patients in the adolescent study (92.4%) and adult study (93.2%) reported at least one AE (Tables 2 and 3). The most frequently occurring AEs regardless of dose at onset were nasopharyngitis (adolescent study: 29.4%/adult study: 18.2%), insomnia (21.0%/15.5%), aggression (16.0%/15.5%) and upper respiratory tract infection (14.3%/16.2%). The rate of AEs was the highest in patients receiving the 100-mg bid dose of mavoglurant, reflecting, at least in part, the greater duration of time that patients spent at this highest dose.

**Table 2:** Most frequent adverse events* in adolescent patients during the extension period, by last dose before event onset (safety population). *Incidence ≥10% in any group.

Preferred term	Total n (%)	Mavoglurant 25 mg bid n (%)	Mavoglurant 50 mg bid n (%)	Mavoglurant 75 mg bid n (%)	Mavoglurant 100 mg bid n (%)
Adolescent study	N = 119	N = 119	N = 118	N = 116	N = 108
Patients with any adverse event	110 (92.4)	36 (30.3)	41 (34.7)	44 (37.9)	90 (83.3)
Nasopharyngitis	35 (29.4)	7 (5.9)	3 (2.5)	8 (6.9)	25 (23.1)
Insomnia	25 (21.0)	4 (3.4)	10 (8.5)	5 (4.3)	12 (11.1)
Aggression	19 (16.0)	3 (2.5)	3 (2.5)	4 (3.4)	12 (11.1)
Initial insomnia	18 (15.1)	3 (2.5)	2 (1.7)	4 (3.4)	9 (8.3)
Upper respiratory tract infection	17 (14.3)	3 (2.5)	5 (4.2)	3 (2.6)	9 (8.3)
Anxiety	15 (12.6)	3 (2.5)	1 (0.8)	0	11 (10.2)
Headache	15 (12.6)	4 (3.4)	1 (0.8)	4 (3.4)	8 (7.4)
Irritability	15 (12.6)	3 (2.5)	1 (0.8)	3 (2.6)	9 (8.3)
Vomiting	13 (10.9)	2 (1.7)	5 (4.2)	0	8 (7.4)
Cough	12 (10.1)	1 (0.8)	1 (0.8)	4 (3.4)	7 (6.5)
Diarrhea	12 (10.1)	2 (1.7)	4 (3.4)	0	5 (4.6)
Patients with any serious adverse event	4 (3.4)	0	1 (0.8)	0	3 (2.8)
Patients with any adverse event leading to study drug discontinuation	6 (5.0)	2 (1.7)	2 (1.7)	0	3 (2.8)

**Table 3:** Most frequent adverse events* in adult patients during the extension period, by last dose on, or before, event onset (safety population).

Preferred term	Total n (%)	Mavoglurant 25 mg bid n (%)	Mavoglurant 50 mg bid n (%)	Mavoglurant 75 mg bid n (%)	Mavoglurant 100 mg bid n (%)
Adult study	N = 148	N = 147	N = 148	N = 141	N = 135
Patients with any adverse event	138 (93.2)	49 (33.3)	47 (31.8)	50 (35.5)	112 (83.0)
Nasopharyngitis	27 (18.2)	2 (1.4)	4 (2.7)	3 (2.1)	21 (15.6)
Upper respiratory tract infection	24 (16.2)	4 (2.7)	1 (0.7)	3 (2.1)	16 (11.9)
Aggression	23 (15.5)	6 (4.1)	8 (5.4)	6 (4.3)	12 (8.9)
Insomnia	23 (15.5)	4 (2.7)	3 (2.0)	7 (5.0)	12 (8.9)
Headache	21 (14.2)	4 (2.7)	3 (2.0)	7 (5.0)	14 (10.4)
Vomiting	18 (12.2)	1 (0.7)	2 (1.4)	2 (1.4)	14 (10.4)
Anxiety	16 (10.8)	1 (0.7)	3 (2.0)	3 (2.1)	10 (7.4)
Cough	16 (10.8)	4 (2.7)	0	2 (1.4)	9 (6.7)
Agitation	15 (10.1)	0	1 (0.7)	3 (2.1)	12 (8.9)
Irritability	15 (10.1)	0	7 (4.7)	4 (2.8)	6 (4.4)
Patients with any serious adverse event	7 (4.7)	1 (0.7)	0	1 (0.7)	6 (4.4)
Patients with any adverse event leading to study drug discontinuation	25 (16.9)	5 (3.4)	5 (3.4)	5 (3.5)	12 (8.9)

*Incidence ≥10% in any group.

AEs were predominantly mild or moderate in severity for both the adolescent study (86%) and adult study (77%). Four patients in the adolescent study (3.4%) and seven in the adult study (4.7%) experienced an SAE. SAEs in the adolescent study were one case each of aggression, hip joint dislocation with subsequent respiratory tract infection as a complication of surgery, foreign body ingestion, and appendicitis. The incident of severe aggression was considered by the investigators as possibly related to study medication, whereas the other three SAEs were considered unrelated.

SAEs in the adult study were: five patients with one or more psychiatric disorder symptoms (aggression, anxiety, agitation, panic attack, and visual and auditory hallucinations) and one patient each with epilepsy/seizure and liver enzyme elevations. SAEs of aggression and visual hallucinations in one patient, aggression in a second patient, and liver enzyme elevations in a third patient were considered possibly related to study drug. All other SAEs were considered unrelated.

Six patients in the adolescent study (5.0%) and 25 in the adult study (16.9%) discontinued study treatment due to an AE. The most frequently reported class of AEs leading to discontinuation were psychiatric disorders (adolescent study: 4.2%/adult study: 12.2%) and nervous system disorders including dizziness, speech disorder, headache, incoherent/poor quality sleep, and psychomotor hyperactivity (adolescent study: 2.5%/adult study: 4.1%). No deaths were reported in either study.

### Clinical effect

#### ABC-C_FX_

**Figure 2:**
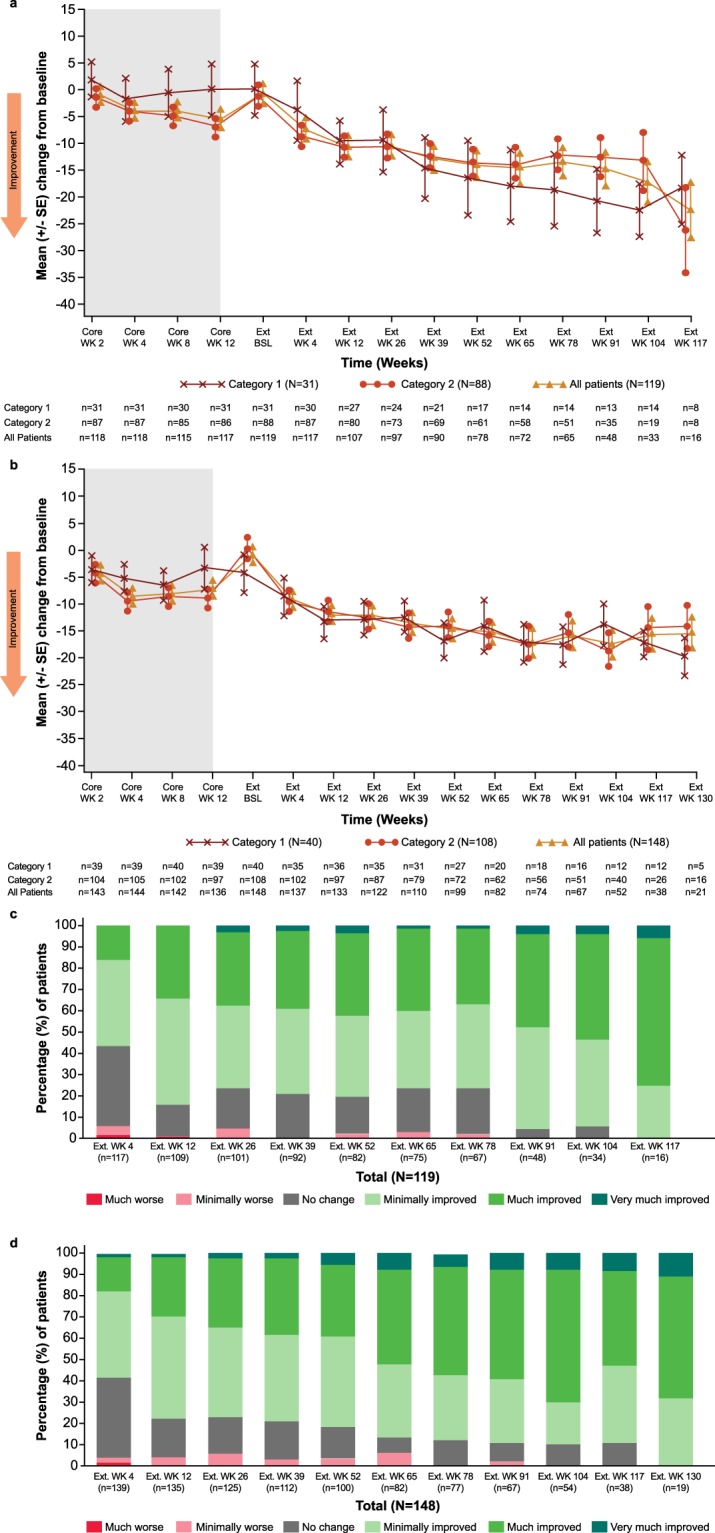
ABC-C_FX_ LS Mean Change from core study baseline in (**a**) adolescents and (**b**) adults and distribution of CGI-I ratings from extension study baseline in (**c**) adolescents and (**d**) adults*. Category 1: treated with mavoglurant in core study and entered the open-label extension study within 1 week of core study completion. Category 2: treated with placebo in core study or entered the open-label extension study more than 1 week after core study completion. Ext, extension; SE, standard error; WK, Week. *All patients regardless of methylation status.

Two categories of patients were analyzed separately, those who received a relatively continuous mavoglurant treatment from the core study, and those who did not (i.e. coming from the placebo arm or with a larger treatment gap; see Fig. 2 for more details).

In both studies and both patient categories, a gradual improvement in the behavioral symptoms of FXS, as measured by a reduction in mean ABC-C_FX_ total scores, was observed from the core study baseline (Fig. 2a,b). The mean (±SD) overall change in ABC-C_FX_ total score from the extension baseline to Week 52 was −13.4 (±22.1) and −13.3 (±19.2) in the adolescent and adult studies, respectively.

#### CGI-I

The distribution of patient CGI-I ratings over time are summarized in Fig. 2c and d and detailed in Table S3. At 12 weeks, clinicians rated the majority of patients (>75%) in both studies as showing an improvement in their global FXS symptoms from the extension study baseline. A continued improvement in CGI-I score distribution was observed over the duration of the extension study. Few patients (n =≤ 6 at any time point) achieved a CGI-I rating of “very much improved” or of “minimally worse,” “much worse” or “very much worse.”

#### RBS-R and SRS

**Figure 3:**
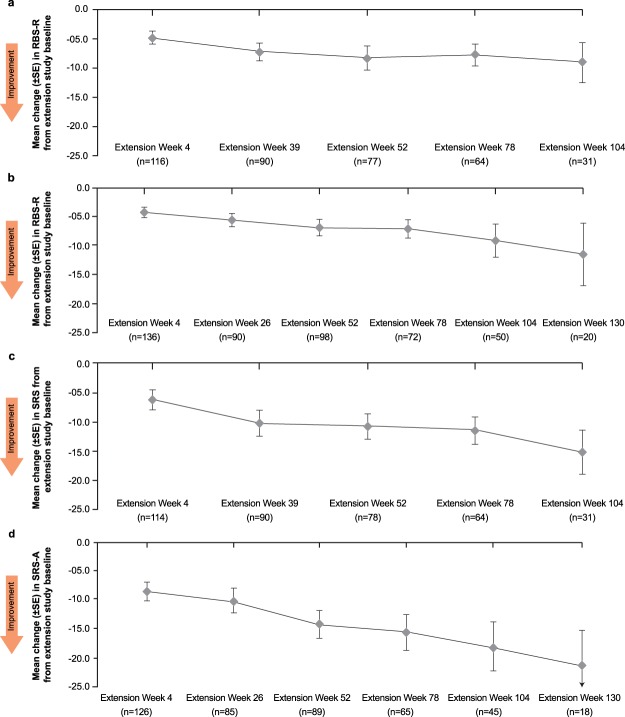
RBS-R score from extension study baseline in (**a**) adolescents and (**b**) adults* and SRS score from extension study baseline in (**c**) adolescents and (**d**) adults*. RBS-R, Repetitive Behavior Scale – Revised; SE, standard error; SRS, Social Responsiveness Scale. *All patients regardless of methylation status.

Patients’ repetitive behavior and social interactions, as measured by the RBS-R and SRS scales, respectively, also showed a gradual improvement from the extension study baseline (Fig. 3 and Table S4).

## Discussion

The primary objective of these open-label, single-arm, extension studies was to evaluate the long-term safety of mavoglurant in adolescents or adults with FXS. Although the studies were terminated prematurely, the extent of study drug exposure provides considerable insight into the long-term safety and tolerability profile of mavoglurant.

The results showed that in adolescent (age 12–19 years) and adult (age 18–45 years) patients with FXS, long-term treatment with mavoglurant was generally well tolerated with low rates of SAEs and no new safety findings beyond those observed in the 12-week double-blind core studies of adolescents or adults. Given the long duration of the treatment period, the majority of patients experienced at least one AE but these events were predominantly mild or moderate in severity and the majority were considered by the investigator to be unrelated to the drug. More patients showed aggressive behavior on the 100 mg dose (11.1% and 8.9% in the adolescent and adult studies, respectively). This could be the result of the longer exposure to this dose rather than a dose effect; in any case future treatment should be carefully monitored and the dose should be lowered if activation and/or aggression occur. Overall, 11 patients experienced an SAE, including four patients for whom the SAEs were possibly related to mavoglurant treatment.

No notable differences in the safety profile of mavoglurant were observed between adolescent and adult patients. Mean exposure to mavoglurant was lower in the adolescent versus the adult study (167.2 patient-years vs. 218.1 patient-years), and a lower proportion of patients in the adolescent study received the intended mavoglurant 100-mg bid dose for ≥1 day (76.5% vs. 91.2%). These differences are likely to be due to the smaller size and body weight of adolescent patients and consequent relative greater exposure at an equivalent dose level. Moreover, the lower number of patients enrolled in the adolescent study, as well as the later start date with an overall shorter study duration relative to the adult study (~34 vs 37 months) could affect patient-years.

Both studies showed a gradual and consistent improvement in FXS behavioral symptom control as measured by ABC-C_FX_ and CGI-I scores. These improvements over the extension baseline were apparent on all behavioral measures without loss of effect in a subgroup over time. The level of improvement in terms of mean (±SD) change in ABC-C_FX_ total scores from extension baseline after 52 weeks of mavoglurant treatment in the extensions studies (−13.4 [±22.1] and −13.3 [±19.2] in adolescents and adults, respectively) was numerically higher than that observed with placebo at 12 weeks in the core studies (−6.2 [ ±17.86] and −10.9 [±15.61], respectively) (data on file)^17^. Trends toward improvement in RBS-R and SRS scores were also observed in the open-label extension studies.

These positive trends should be interpreted with caution given that the studies do not have a control group and thus cannot make comparative assessment of efficacy. It would be expected that placebo effects would taper off after such a long period, however there is some data to indicate that placebo responses are stable over time (up to three years) in populations with developmental disorders^25^. Other possible limitations of the study designs include bias associated with open-label treatment and selection/survivor bias due to enrollment of patients who successfully completed prior core studies of mavoglurant. In addition, a relatively large proportion of patients discontinued study treatment before study termination (24.4% in the adolescent study and 46.6% in the adult study), including 17 (14.3%) and 35 (23.6%) patients, respectively, due to a perceived lack of efficacy. Thus, an ongoing improvement on three behavioral measures over the course of the study may also result from the selection of those patients perceived to be doing better independent of a drug effect rather than reflecting a subgroup of long-term responders. For all these reasons it was not deemed appropriate to test whether the observed improvement over the core study was statistically significant.

In any case, the above interpretation of the results does not preclude the possibility of mavoglurant being effective in a different experimental setting (younger age group, longer trial periods to assess cognition and development, applying interventional measures such as speech/reading training, or adding a drug with a synergistic mechanism of action). It should also be noted that, while many families observed an improvement in language, focus and aptitude to get things done, job performance and problem-solving abilities, possible effects on cognition were not assessed. Although work in this area is ongoing, there is no consensus yet on which specific measures should be used to evaluate population with moderate or severe intellectual disabilities, who often perform at or below the floor of standardized cognitive test^26^. Future studies of mavoglurant should evaluate cognitive benefits and initiate treatment at a younger age when antagonism of the mGluR pathway is more likely to facilitate normalization of brain development in children with FXS.

The FXS clinical trial field has undergone significant growth in recent years, and its future, including the future of the *fmr1* KO mouse model and more generally the translation of preclinical research, have been discussed in recent reviews^26–29^. Assessment measures including electroencephalogram (EEG) recordings and functional magnetic resonance imaging (fMRI), which can be applied in preclinical models as well as clinically, could help bridge the translation treatment gap^26,27^. Altogether, such studies would shed some light on the current disconnect between the restorative preclinical effects of mGluR5 blockers repeatedly and extensively observed in animal models, and the lack of significant clinical effect observed in humans.

## Electronic supplementary material

Supplementary Files
