# Supplementary material for: Mavoglurant in Fragile X Syndrome: Results of two open-label, extension trials in adults and adolescents
Source: Sci Rep. 2018 Nov 19;8:16970. doi: 10.1038/s41598-018-34978-4 (PMC6242849; doi:10.1038/s41598-018-34978-4)
Supplement: Supplementary file 1 — Supplementary Files [file 41598_2018_34978_MOESM1_ESM.pdf]

Title: Mavoglurant in Fragile X Syndrome: Results of two open-label, extension trials in adults and adolescents

Authors

Randi Hagerman[1], Sebastien Jacquemont[2, 3], Elizabeth Berry-Kravis[4], Vincent Des Portes[5], Andrew Stanfield[6], Barbara Koumaras[7], Gerd Rosenkranz[8], Alessandra Murgia[9], Christian Wolf[10], George Apostol[8], and Florian von Raison*[8]

Affiliations

[1]MIND Institute and Department of Pediatrics, UC Davis Medical Center, Sacramento, CA, USA, [2]Centre Hospitalier Universitaire Vaudois, Lausanne, Switzerland, [3]CHU Sainte-Justine Research Centre, Montreal, Canada, [4]Rush University Medical Centre, Department of Pediatrics, Neurological Sciences, and Biochemistry, Chicago, IL, USA, [5]National Reference Center for Fragile X and Other XLID, CIC 1407 INSERM - Hospices Civils de Lyon, Université de Lyon and CNRS UMR 5304 (L2C2), Bron, France, [6]Patrick Wild Centre, Division of Psychiatry, University of Edinburgh, Royal Edinburgh Hospital, Edinburgh, UK, [7]Neurodegeneration Global Development, Novartis Pharmaceuticals Corporation, East Hanover, NJ, USA, [8]Neuroscience Development, Novartis Pharma AG, Basel, Switzerland, [9]Laboratory of Molecular Genetics of Neurodevelopment, Department of Women's and Children's Health, University of Padova, Padova, Italy, [10]Lycalis sprl, Brussels, Belgium

Corresponding author

*Florian von Raison

Neuroscience Development, Novartis Pharma AG, Postfach, CH 4002 Basel, Switzerland

Tel: +41797998993

Fax: +41 61 696 8176

Email: florian.von_raison@novartis.com

Text summary:

Table S1: List of Independent Ethics Committees (IEC) or Institutional Review Boards (IRB) by study centre for the adolescent study (NCT01433354)

Table S2: List of Independent Ethics Committees (IEC) or Institutional Review Boards (IRB) by study centre for the adult study (NCT01348087)

Table S3: Distribution of all patient CGI-I ratings from extension study baseline

Table S4: Mean (±SD) RBS-R and SRS total scores of all patients from extension study baseline

Figure S1: Study design

Supplemental tables and figures

Table S1: List of Independent Ethics Committees (IEC) or Institutional Review Boards (IRB) by study centre for the adolescent study (NCT01433354)

Center No.	Ethics Committee or Institutional Review Board	Department / Organization	City, State/Province, Postal Code Country
0121	Children's Hospitals Network Human Research Ethics Committee	Child Department Unit	Westmead Sydney 2145 Australia
0122	Children's Hospitals Network Human Research Ethics Committee	Child Department Unit	Melbourne Victoria 3052 Australia
0201	Université Catholique de Louvain – Faculté de Médecine	Commission d'Ethique Biomédicale Hospitalo-Facultaire	Brussels 1200 Belgium
0202	UZ Leuven	Commissie Medische Ethiek - Toetsingscommissie	Leuven 3000 Belgium
0161	De Videnskabsetiske Komiteer for Region Hovedstaden	Kongens Vænge 2	Hillerød 3400 Denmark
0361	CPP Sud-Est- II - LYON	Groupe Hospitalier Edouard Herriot - BAT 12 – 1°étage	Lyon 69437 France
0241	Ethik-Kommission	Der Medizinischen Fakultät der Eberhard-Karls-Universität und am Universitätsklinikum Tübingen	Tübingen 72074 Germany
0243	Ethik-Kommission	Der Landesärztekammer Rheinland-Pfalz	Mainz 55166 Germany
0245	Ethik-Kommission	Der Medizinischen Fakultät der Eberhard-Karls-Universität und am Universitätsklinikum Tübingen	Tübingen 72074 Germany
0131	The Haim Sheba MC Ethic Committee	Tel Hashomer	Ramat Gan 52621 Israel
0402	Comitato Etico Dell'Azienda Ospedaliera Universitaria San Martino Di Genova	N/A	Genova GE 16132 Italy
0403	Comitato Etico Per Le Sperimentazioni Cliniche Della Provincia Di Padova Presso Aou	N/A	Padova 35128 Italy
0481	Medisch Ethische	N/A	Rotterdam 3000 CA

	Toetsings<br>Commissie Erasmus MC		Netherlands
0211	CEIC Corporació Sanitaria<br>Parc Taulí	Fundació Parc Taulí	Sabadell Barcelona 08208<br>Spain
0212	Hospital General de<br>Catalunya	N/A	San Cugat del Vallès Barcelona<br>08190<br>Spain
0213	Comité Ético & Gestión<br>Económica	Hospital R.U. Carlos Haya	Malaga Andalucia 29010<br>Spain
0721	Regionala<br>etikprövningsnämnden i<br>Stockholm	N/A	Stockholm FE 289 171 77<br>Sweden
0281	Kantonale<br>Ethikkommission<br>Zurich (KEK)	N/A	Zurich Zurich 8090<br>Switzerland
0282	Commission cantonale<br>d'éthique de la recherche<br>sur l'être humain	N/A	Lausanne Canton de Vaud 1012<br>Switzerland
0321	Scotland A Regional<br>Ethical<br>Committee	N/A	Edinburgh Scotland EH1 3EG<br>United Kingdom
0561	UC Davis Institutional<br>Review Board	N/A	Sacramento California 95817<br>United States
0562	Emory University<br>Institutional Review Board	N/A	Atlanta Georgia 30322<br>United States
0564	Childrens Hospital Boston	Childrens Hospital<br>Investigations	Boston Massachusetts 02115<br>United States
0565	University of Nebraska<br>Medical Center<br>Institutional Review Board	Office of Regulatory Affairs	Omaha Nebraska 68198-7830<br>United States
0566	New York state Psychiatric<br>Institute Institutional<br>Review<br>Board	N/A	Riverside Drive New York 10032<br>United States
0567	Rush office of the<br>Research<br>and Clinical Trials<br>Administration	N/A	Chicago Illinois 60612<br>United States
0568	Vanderbilt Instituional<br>Review Board	N/A	Nashville Tennessee 37232<br>United States

Table S2: List of Independent Ethics Committees (IEC) or Institutional Review Boards (IRB) by study centre for the adult study (NCT01348087)

Center No.	Ethics Committee or Institutional Review Board	Department / Organization	Address Country
0101	Travel Clinics Australia		Caulfield VIC 3161 Australia
0102	Hunter New England Research Ethics & Governance Unit	Research Ethics & Governance Unit	New Lambton NSW 2305 Australia
0103	Hunter New England Research Ethics & Governance Unit	Research Ethics & Governance Unit	New Lambton NSW 2305 Australia
0421	IRB Services		Aurora ON L4G 0A5 Canada
0423	Centre Hospitalier Universitaire de Sherbrooke		Sherbrooke QC J1H 5N4 Canada
0141	Videnskabsetisk Komité for Region Hovedstaden		Hillerød Denmark 3400 Denmark
0341	CPP SUD-EST II	Hopital Edouard Herriot Batiment 12 - 1er étage	Lyon 69437 France
0342	CPP SUD-EST II	Hopital Edouard Herriot Batiment 12 - 1er étage	Lyon 69437 France
0221	Landesamt Fuer Gesundheit und Soziales Geschaeftsstelle der Ethik-Kommission des Landes Berlin		Berlin Fehrbelliner Platz 10707 Germany
0222	Eberhard Karls Universitat Tubingen Ethik-Kommission an der Medizinschen Fakultaet der Eberhard-Karls-Universitaet und am Universitaetsklinikum Tübingen		Gartenstrasse Tübingen 72074 Germany
0223	Landesaerztekammer Rheinlandpfalz / Ethik Kommission		Mainz Deutschhausplatz 55116 Germany
0224	Ethik-Kommission der Bayerischen Landesarztekammer		Muehlbaurstrasse Muenchen 81677 Germany
0382	Comitato di Etica dell'IRCCS Istituto Giannina Gaslini di		Genova 16132 Italy

	Genova Largo<br>Gerolamo Gaslini		
0201	CEIC idcsalud Hospital<br>General de Catalunya		Sant Cugat del Vallès 08195<br>Spain
0202	CEIC Hospital<br>Regional Universitario<br>Carlos Haya		Málaga 29010<br>Spain
0261	Kantonale<br>Ethikkommission<br>Zurich (KEK)		Zurich 8090<br>Switzerland
0262	Canton de vaud<br>Commission cantonale<br>d'ethique de la<br>recherche sur l'etre<br>humain		Lausanne 1005<br>Switzerland
0301	National Research<br>Ethics Service<br>Scotland A Research<br>Ethics committee		Edinburgh EH1 3EG<br>United Kingdom
0501	Indiana University<br>Institutional Review<br>Board	Office of Research Administration	Indianapolis IN 46202<br>United States
0503	Emory University<br>Institutional Review<br>Board		Atlanta GA 30322<br>United States
0504	UC Davis Institutional<br>Review Board	IRB Administration	Sacramento CA 95817<br>United States
0505	New York State<br>Psychiatric Institute		New York NY 10032<br>United States
0506	University of Nebraska<br>Medical Center<br>Institutional Review<br>Board	UNMC Office of Regulatory Affairs<br>(ORA)	Omaha NE 68198-7830<br>United States
0507	Rush Office of<br>Research and Clinical<br>Trials Administration		Chicago IL 60612<br>United States
0508	Quorum Review , Inc.		Seattle WA 98101<br>United States
0509	Vanderbilt IRB		Nashville TN 37232<br>United States
0510	Quorum Review , Inc.		Seattle WA 98101<br>United States
0513	Boston Children's<br>Hospital	Office of Clinical Investigations<br>Children's Hospital Boston c/o Office of<br>Clinical Investigations	Boston MA 02115<br>United States

Table S3: Distribution of CGI-I ratings from extension study baseline*

Time point	Clinical global impression — improvement score						
	1<br>Very much improved	2<br>Much improved	3<br>Minimally improved	4<br>No change	5<br>Minimally worse	6<br>Much worse	7<br>Very much worse
**Adolescent study (N = 119), n (%)**							
Week 4 (n=117)	0	19 (16.2)	47 (40.2)	44 (37.6)	5 (4.3)	1 (0.9)	1 (0.9)
Week 12 (n=109)	0	37 (33.9)	55 (50.5)	16 (14.7)	1 (0.9)	0	0
Week 26 (n=101)	3 (3.0)	35 (34.7)	39 (38.6)	19 (18.8)	5 (5.0)	0	0
Week 39 (n=92)	2 (2.2)	34 (37.0)	37 (40.2)	19 (20.7)	0	0	0
Week 52 (n=82)	3 (3.7)	32 (39.0)	31 (37.8)	14 (17.1)	2 (2.4)	0	0
Week 65 (n=75)	1 (1.3)	29 (38.7)	27 (36.0)	16 (21.3)	2 (2.7)	0	0
Week 78 (n=67)	1 (1.5)	24 (35.8)	29 (43.3)	12 (17.9)	1 (1.5)	0	0
Week 91 (n=48)	2 (4.2)	21 (43.8)	23 (47.9)	2 (4.2)	0	0	0
Week 104 (n=34)	1 (2.9)	17 (50.0)	14 (41.2)	2 (5.9)	0	0	0
Week 117 (n=16)	1 (6.3)	11 (68.8)	4 (25.0)	0	0	0	0
Week 130 (n=4)	0	2 (50.0)	1 (25.0)	1 (25.0)	0	0	0
Week 143 (n=2)	0	2 (100.0)	0	0	0	0	0
**Adult study (N = 148), n (%)**							
Week 4 (n=139)	1 (0.7)	23 (16.5)	58 (41.7)	52 (37.4)	3 (2.2)	2 (1.4)	0
Week 12 (n=135)	2 (1.5)	38 (28.1)	65 (48.1)	25 (18.5)	5 (3.7)	0	0
Week 26 (n=125)	2 (1.6)	41 (32.8)	54 (43.2)	21 (16.8)	6 (4.8)	1 (0.8)	0
Week 39 (n=112)	2 (1.8)	41 (36.6)	46 (41.1)	20 (17.9)	3 (2.7)	0	0

Week 52 (n=100)	5 (5.0)	34 (34.0)	43 (43.0)	15 (15.0)	3 (3.0)	0	0
Week 65 (n=82)	6 (7.3)	37 (45.1)	28 (34.1)	6 (7.3)	4 (4.9)	1 (1.2)	0
Week 78 (n=77)	4 (5.2)	40 (51.9)	24 (31.2)	9 (11.7)	0	0	0
Week 91 (n=67)	5 (7.5)	35 (52.2)	20 (29.9)	6 (9.0)	1 (1.5)	0	0
Week 104 (n=54)	4 (7.4)	34 (63.0)	11 (20.4)	5 (9.3)	0	0	0
Week 117 (n=38)	3 (7.9)	17 (44.7)	14 (36.8)	4 (10.5)	0	0	0
Week 130 (n=19)	2 (10.5)	11 (57.9)	6 (31.6)	0	0	0	0
Week 143 (n=7)	0	3 (42.9)	3 (42.9)	1 (14.3)	0	00	0
Week 156 (n=2)	0	1 (50.0)	1 (50.0)	0	0	0	0

CGI-I, Clinical global impression — improvement

** All patients regardless of methylation status*

Table S4: Mean (±SD) RBS-R and SRS total scores from extension study baseline*

Time point	RBS-R total score			SRS total score		
	N	Mean (±SD)	Mean (±SD) change from baseline	n	Mean (±SD)	Mean (±SD) change from baseline
**Adolescent study (N = 119)**						
Extension baseline	119	27.7 (20.35)	N/A	116	96.4 (25.96)	N/A
Extension Week 4	116	22.9 (18.07)	–4.8 (11.90)	114	90.0 (27.98)	–6.2 (18.08)
Extension Week 39	90	20.4 (16.69)	–7.3 (14.29)	90	84.5 (26.45)	–10.4 (20.57)
Extension Week 52	77	20.2 (15.51)	–8.3 (17.85)	78	85.8 (25.14)	–10.7 (18.69)
Extension Week 78	64	19.3 (14.53)	–7.8 (15.10)	64	84.7 (25.72)	–11.6 (18.20)
Extension Week 104	31	18.8 (13.46)	–9.1 (18.78)	31	78.6 (28.20)	–15.2 (20.72)
**Adult study (N = 148)**						
Extension baseline	142	25.2 (19.10)	N/A	132	93.2 (28.80)	N/A
Extension Week 4	136	20.0 (17.38)	–4.6 (10.36)	126	84.2 (28.35)	–8.4 (18.21)
Extension Week 26	90	18.8 (14.61)	–5.6 (10.65)	85	83.5 (23.99)	–10.1 (19.55)
Extension Week 52	98	18.1 (15.60)	–7.1 (13.82)	89	80.1 (28.74)	–14.3 (22.78)
Extension Week 78	72	17.2 (13.79)	–7.5 (13.66)	65	78.1 (27.36)	–15.7 (24.79)
Extension Week 104	50	16.8 (15.95)	–9.4 (20.14)	45	77.0 (30.23)	–18.1 (28.50)
Extension Week 130	20	12.3 (11.58)	–11.6 (23.92)	18	64.3 (31.76)	–21.0 (23.44)

N/A, not applicable; RBS-R, Repetitive Behavior Scale — Revised; Social responsiveness scale; SD, standard deviation

** All patients regardless of methylation status*

Figure S1: Study design

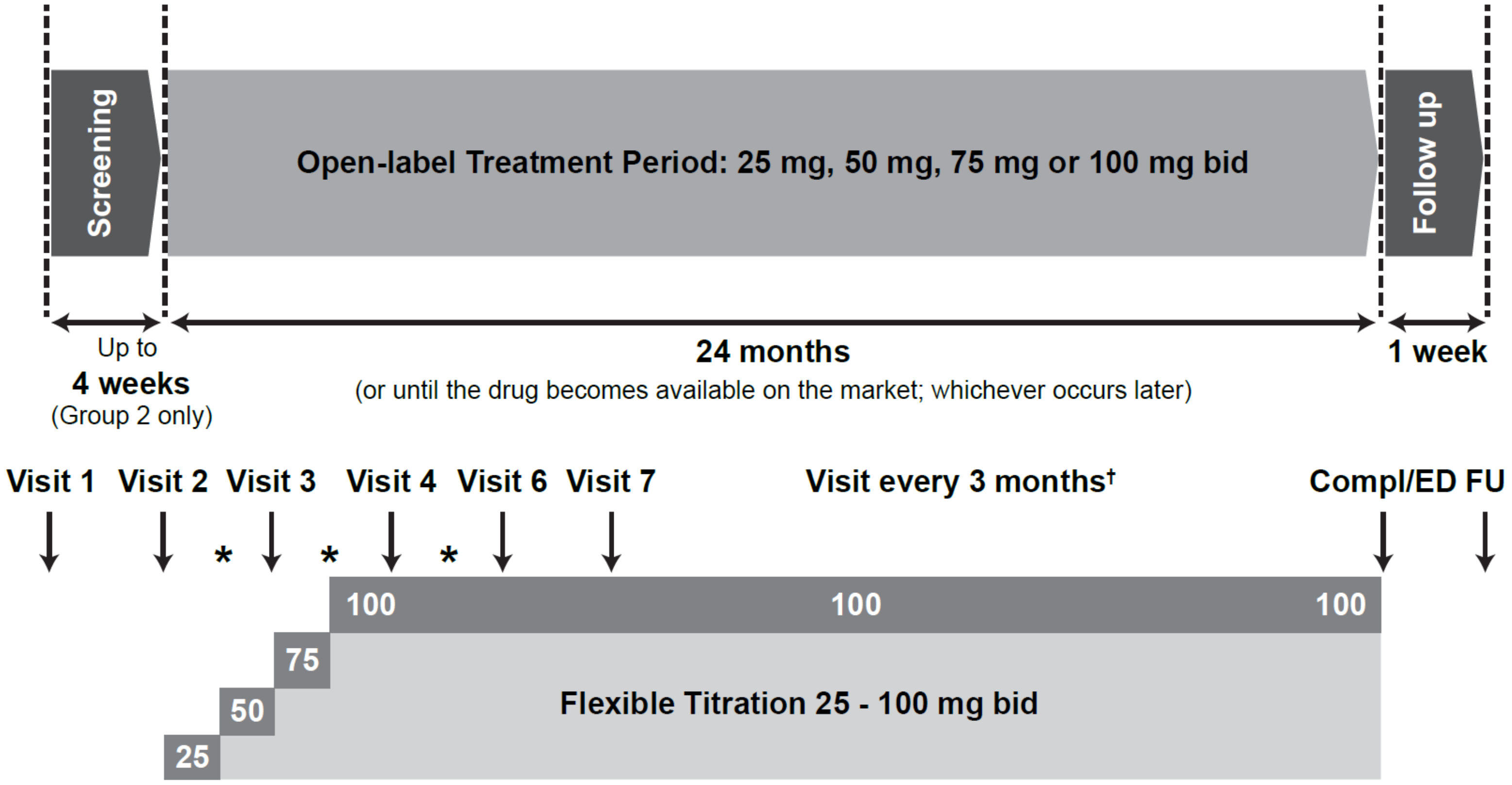

bid, twice daily; Compl, completion; ED, early discontinuation; FU, follow up

***** *Telephone contact visits*

†Treatment was planned to be provided for 24 months or until the study drug became available on the market (whichever occurred later); study visits were to be extended beyond 24 months, if required.

Group 2: patients who entered the extension study more than 1 week after core study completion (distinct from the term "Category" used for presentation of the data)
